# Influence of Hydrogen-Based Direct Reduction Shaft Furnace Interior Structure on Shaft Furnace Performance

**DOI:** 10.3390/ma18204794

**Published:** 2025-10-20

**Authors:** Qingbin Xue, Haotian Liao, Jianliang Zhang, Kejiang Li

**Affiliations:** 1 School of Metallurgical and Ecological Engineering, University of Science and Technology Beijing, 30 Xueyuan Rd., Haidian District, Beijing 100083, China; 13911776225@139.com (Q.X.); liaohtian@163.com (H.L.); 2 School of Chemical Engineering, The University of Queensland, St Lucia, QLD 4072, Australia

**Keywords:** hydrogen-based shaft furnace, DEM, diverter device, particle flow behavior

## Abstract

Hydrogen-based direct reduction of iron ore is a promising route to reduce CO_2_ emissions in steelmaking, where uniform particle flow inside shaft furnaces is essential for efficient operation. In this study, a full-scale three-dimensional Discrete Element Method (DEM) model of a shaft furnace was developed to investigate the effects of a diverter device on granular flow. By systematically varying the radial width and top/bottom diameters of the diverter, particle descent velocity, residence time, compressive force distribution, and collision energy dissipation were analyzed. The results demonstrate that introducing a diverter effectively suppresses funnel flow, prolongs residence time, and improves radial flow uniformity. Among the tested configurations, the smaller central diameter diverter showed the most favorable performance, achieving a faster and more uniform descent, reduced compressive force concentration, and lower collision energy dissipation. These findings highlight the critical role of diverter design in regulating particle dynamics and provide theoretical guidance for optimizing shaft furnace structures to enhance the efficiency of hydrogen-based direct reduction processes.

## 1. Introduction

The steel industry is a central driver of global industrialization, yet it also represents one of the major sources of carbon emissions. The conventional blast furnace–basic oxygen furnace (BF–BOF) route remains dominant, relying on coke as both a reducing agent and a permeability-supporting medium, which results in high energy consumption and significant pollution. Statistics indicate that the steel sector accounts for approximately 7% of global anthropogenic carbon dioxide emissions, with China’s steel industry alone contributing up to 15% of the nation’s total emissions, highlighting the urgent need for decarbonization [1,2,3]. This environmental pressure has driven the global pursuit of low-carbon alternative technologies. Direct reduced iron (DRI) processes, particularly hydrogen-based direct reduction in shaft furnaces, offer a revolutionary approach by substituting hydrogen for carbon-based reductants. Hydrogen reduces iron oxides to produce water vapor instead of carbon dioxide, thereby significantly lowering the carbon footprint [4,5]. For instance, projects such as HYBRIT have successfully established pilot plants, demonstrating the feasibility of hydrogen-based DRI [6]. However, direct reduction shaft furnaces constitute highly complex, multi-scale solid–fluid systems, involving the coupled interactions of gas–solid reaction kinetics, thermodynamics, and particle flow, and their industrial-scale implementation still faces considerable challenges [7,8].

As the core reactor for DRI production, the performance of hydrogen-based shaft furnaces is highly dependent on internal flow uniformity. These furnaces feature a top-wide, bottom-narrow geometry, where particles such as pellets descend under gravity and react countercurrently with ascending reducing gases, primarily hydrogen. This design tends to induce non-uniform particle flow: particles in the central region descend too quickly, forming funnel flow, while particles near the walls experience prolonged residence time, leading to uneven reduction and localized overheating [9]. For example, during furnace scale-up, central material may remain insufficiently reduced, while peripheral material becomes over-reduced, resulting in iron content variations of up to 20%, severely affecting metallurgical efficiency and production stability [10]. Such non-uniformity not only increases energy consumption but also reduces hydrogen utilization efficiency [11]. The non-uniform flow and segregation of particles may result in inefficient gas-solid contact and reduced reduction efficiency, thereby increasing energy consumption and carbon emissions [12]. To optimize flow, industrial applications have introduced structural components such as flow distributors and loosening devices; however, their regulatory mechanisms under hydrogen-based conditions have not yet been systematically quantified [13,14].

Research on direct reduction shaft furnaces has primarily focused on two areas: reaction kinetics and system modeling. In terms of reaction kinetics, significant progress has been made in understanding the microscopic mechanisms of hydrogen reduction of iron oxides. Turkdogan and Vinters [15] experimentally verified the effect of particle size on reduction rates, showing that smaller particles reduce 40% faster than larger ones. Zielinski et al. [16] analyzed the influence of the H_2_/H_2_O ratio on reaction thermodynamics, finding that higher hydrogen content enhances reduction efficiency but exacerbates particle swelling. Pineau et al. [17,18] investigated the effect of temperature on nucleation and phase-boundary reactions, demonstrating that reaction rates increase exponentially above 800 °C. Furthermore, particle swelling, as a critical challenge, was studied by Zhao et al. [19], who examined the effects of temperature and concentration and proposed mitigating swelling through optimization of pellet basicity. While these studies have deepened the understanding of hydrogen reduction mechanisms, they have largely focused on laboratory-scale experiments and have not fully addressed flow–reaction coupling at an industrial scale [20].

In the field of modeling, the discrete element method (DEM) and continuum models based on computational fluid dynamics (CFD) are the primary tools. DEM, introduced by Cundall and Strack [21], provides particle-level insights, such as particle trajectories, velocities, and collision energies. For instance, Boechat et al. [22] applied DEM to simulate local regions of shaft furnaces, revealing the influence of wall friction on flow patterns. However, DEM is computationally expensive and difficult to apply to full-scale commercial furnaces containing billions of particles, and is therefore often limited to scaled-down models [13,14]. In contrast, continuum models, which are based on the conservation equations of mass, momentum, and energy, are more suitable for industrial-scale simulations. Costa et al. [23] developed a two-dimensional shaft furnace model to predict temperature and gas-phase distributions, while Hamadeh et al. [8] further integrated eight heterogeneous reactions to optimize the design of the reduction zone. Although these models are computationally efficient, they lack particle-scale resolution and are unable to capture microscale flow heterogeneities [24].

Although continuum models demonstrate high efficiency in industrial-scale shaft furnace simulations, their inherent scale limitations prevent accurate resolution of microscale flow heterogeneities [8,24]. While DEM can address this limitation [21,25], existing studies are constrained by computational costs and have predominantly focused on proportionally scaled-down models [13,14], localized region analyses [22], or increased particle diameters [26]. To date, a full-scale DEM model corresponding strictly to the structure and operating conditions of a commercial shaft furnace has not been established.

In particular, there is a lack of comprehensive understanding regarding the effects of internal structural features—such as the geometry of diverter devices—on particle flow trajectories, velocity distributions, and force chains within hydrogen-based shaft furnaces. This gap has limited the ability to optimize furnace design for uniform particle flow and efficient reduction under hydrogen-based conditions.

Therefore, the present study establishes a three-dimensional DEM model based on the MIDREX shaft furnace structure. Under continuous feed/discharge conditions, the model is employed to systematically investigate the influence of diverter device geometry on furnace performance. The novelty of this work lies in quantifying, how variations in diverter shape and size affect particle flow trajectories, bed descent velocity, and particle force conditions in an industrial-scale hydrogen-based shaft furnace. These findings provide theoretical guidance for optimizing internal structures to improve hydrogen utilization efficiency and reduction performance.

## 2. Methodology

### 2.1. Discrete Element Method

The discrete element method (DEM), pioneered by Cundall and Strack [21], employs Newtonian mechanics to simulate granular dynamics. DEM has been widely used in the study of granular flow behavior due to its ability to capture particle-particle interactions and the dynamic evolution of particle configurations. The mechanical behavior of a particle *i* is governed by the following equations:

(1) $$m_{i}\frac{dv_{i}}{dt}=\sum_{j=1}^{k_{i}}(F_{cn,ij}+F_{dn,ij}+F_{ct,ij}+F_{dt,ij})+m_{i}g$$

(2) $$I_{i}\frac{d\omega_{i}}{dt}=\sum_{j=1}^{k_{i}}(T_{t,ij}+T_{r,ij})$$

Particle dynamics are governed by its intrinsic properties, including mass (***m_i_***) and velocity (***v_i_***), and its interactive environment, defined by the number of contacting particles (***k_i_***). The normal contact forces (***F_cn,ij_***), normal damping forces (***F_dn,ij_***), tangential contact force (***F_ct,ij_***), tangential damping forces (***F_dt,ij_***), and gravitational force (***m_i_g***) are also considered. Rotational dynamics are governed by the moment of inertia (***I_i_***) and angular velocity (***ω_i_***), with torques arising from tangential forces (***T_t,ij_***) and rolling friction resistance (***T_r,ij_***). The net force and torque on each particle are obtained through vector summation of all interactive components. Comprehensive formulations for these interactions are available in existing literature [13,27,28,29].

### 2.2. Simulated Setup

**Figure 1:**
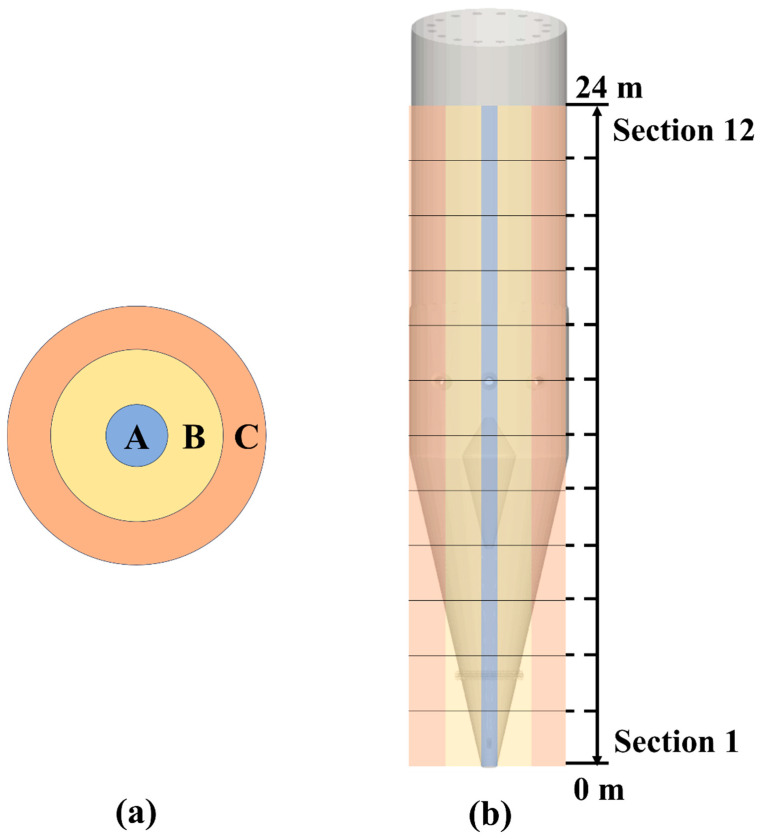
Schematic of shaft furnace region divisions: (**a**) radial divisions, (**b**) longitudinal divisions.

A 3D model of an industrial shaft furnace, with a height of approximately 27 m and a diameter of about 5.7 m, was established and investigated using the open-source DEM software LIGGGHTS (version 3.X). As shown in Figure 1, the furnace was divided radially into three regions and longitudinally into twelve sections, with detailed partitioning presented in Table 1. The reference furnace dimensions were determined based on previous studies [22], and Table 2 lists the material properties and simulation parameters used in this study [13,14,22]. To reduce computational costs, the pellet size was increased several times compared to actual dimensions [26] in all cases, the pellet diameter was set to 96 mm. Importantly, the DEM material property parameters adopted in this study were strictly selected in accordance with previously validated works [13,14,22]. These studies have confirmed that such parameter settings can reliably reproduce realistic particle flow behavior in shaft furnaces and hoppers, thereby ensuring the credibility of the present simulation. Moreover, although the pellet size was enlarged for computational efficiency, this approach is widely employed in large-scale DEM studies [25]. The enlargement influences only the absolute velocity scale but does not alter the relative flow trends, such as the mitigation of central funnel flow by the diverter or the effect of distributor diameter on flow uniformity. Hence, the principal conclusions of this study are not affected by the particle size scaling.

In the present simulations, approximately 640,000 particles were modeled, with a time step of 1 × 10^−5^ s. Each case was executed using 64 CPU cores for about 48 h. At the start of the discharge process simulation, new particles were introduced into the furnace through 16 feed inlets located at the top, and exited from the bottom of the shaft furnace.

**Figure 2:**
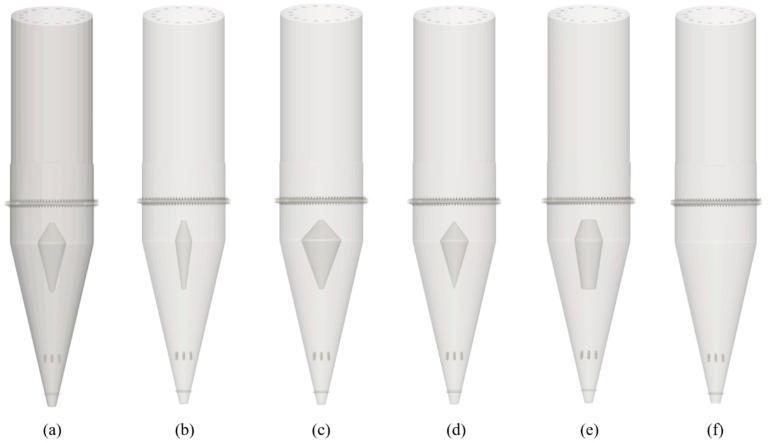
3D models of the shaft furnace with different flow distributor sizes and shapes: (**a**) Case 1, (**b**) Case 2, (**c**) Case 3, (**d**) Case 4, (**e**) Case 5, (**f**) Case 6.

Figure 2 presents 3D models of the shaft furnace with different sizes and shapes of flow distributors. The shaft furnace has an inner diameter of approximately 5.7 m in the reduction zone and a total height of about 27 m, including a conical section of 11.4 m in height. The furnace outlet has a diameter of about 0.57 m. To improve material flow, three loosening devices were installed along the shaft: the upper and middle devices each consist of three loosening rods, whereas the lower device consists of a single rod. All loosening devices operate at a fixed angle and period, alternating between clockwise and counterclockwise rotation within each cycle.

In Figure 2a, the baseline furnace configuration was used to adjust the distributor dimensions, with specific adjustments detailed in Table 3. The diverter device has an upper height of approximately 1.4 m and a lower height of about 3.4 m, while the other geometric parameters, such as central diameter and top/bottom diameter, are listed in Table 3 for all investigated cases.

## 3. Results and Discussion

### 3.1. Analysis of the Effects on Particle Flow Velocity

**Figure 3:**
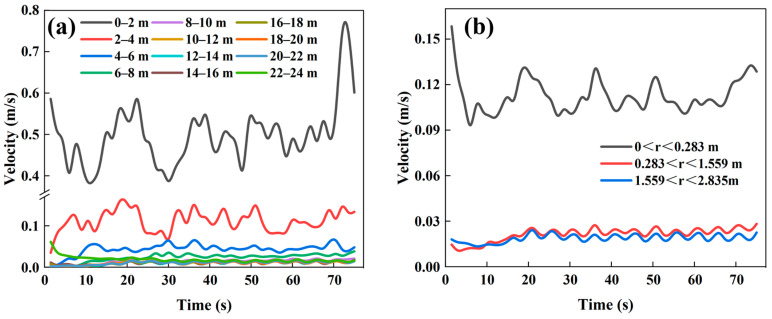
Temporal variation in average particle descent velocity in different regions under the baseline case (Case 1): (**a**) longitudinally divided regions, (**b**) radially divided regions.

**Figure 4:**
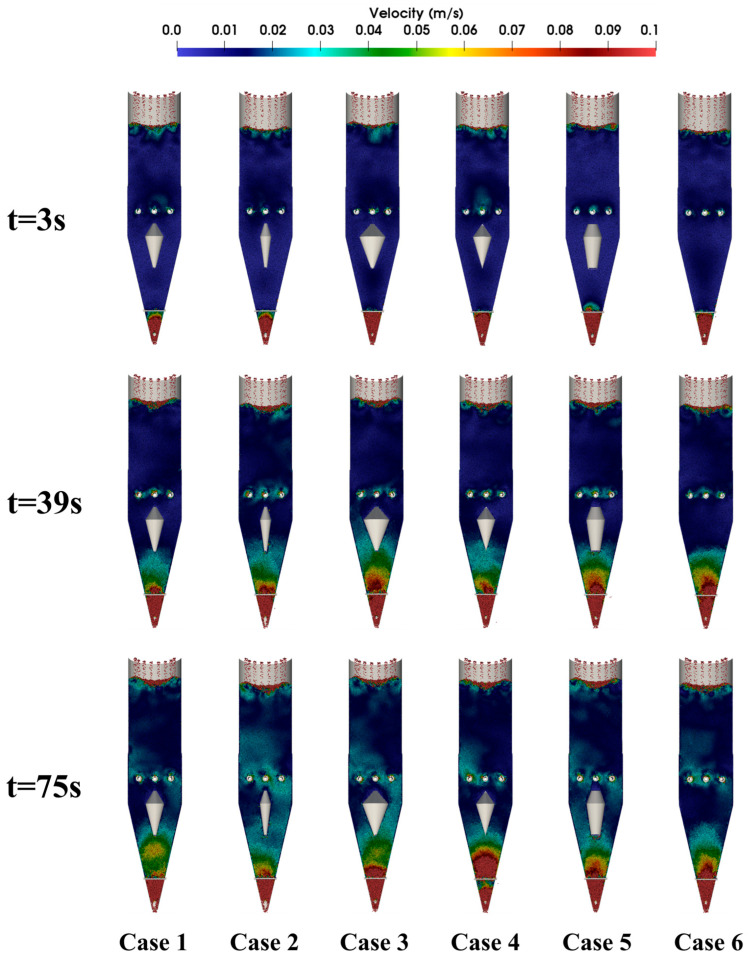
Contour maps of particle descent velocity within the shaft furnace at different times.

Figure 3 shows the temporal variation in the average particle descent velocity in different regions of the baseline furnace (Case 1). It should be noted that within the height range of 8–24 m inside the shaft furnace, the differences in particle descent velocity among regions are very small, which is also confirmed by the velocity contour plots in Figure 4. The variations in particle descent velocity across longitudinal and radial regions reflect the flow uniformity and the dynamic behavior of particles within the furnace. As shown in Figure 3a, the particle descent velocity decreases with increasing height, with significant fluctuations observed in the 0–2 m height range. When the height exceeds 4 m, the temporal variation in the average particle descent velocity becomes more stable, and the velocity differences between regions are reduced.

In the radially divided regions (Figure 3b), particles in the central region descend more rapidly, exhibiting funnel flow, whereas particles near the walls descend more slowly. In addition, the temporal fluctuations in the average particle descent velocity are larger in the furnace center, while the differences in average velocities between the sub-central and peripheral regions are smaller. Around t =20 s, the average velocities in these two regions stabilize, indicating that the particle descent velocity within the furnace has reached a dynamic equilibrium.

**Figure 5:**
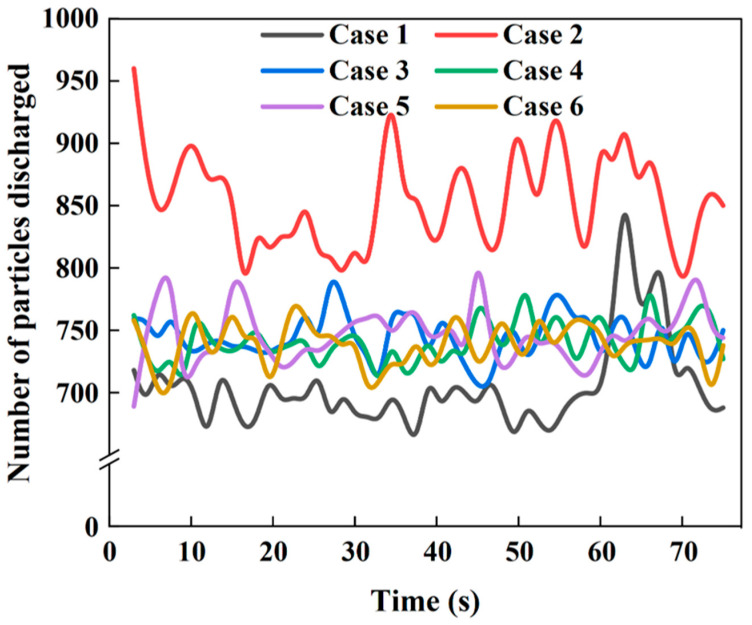
Temporal variation in particle discharge from the shaft furnace.

Figure 4 presents velocity contour maps of particle descent within the shaft furnace at different times. The velocity distribution can be distinguished according to the color scale, where warmer colors correspond to higher descent velocities. It can be observed that, in all cases, particle descent velocity increases with decreasing height, reaching a peak near the furnace outlet. At t = 3 s, the particle flow inside the furnace has not yet stabilized; only particles near the outlet exhibit relatively high descent velocities, while velocities in other regions remain very low and show minimal differences. By t = 39 s, the particle velocity field exhibits significant differentiation, and flow heterogeneity becomes apparent. Figure 5 further shows the temporal variation in particle discharge from the furnace, with Case 2 demonstrating the best flowability.

**Figure 6:**
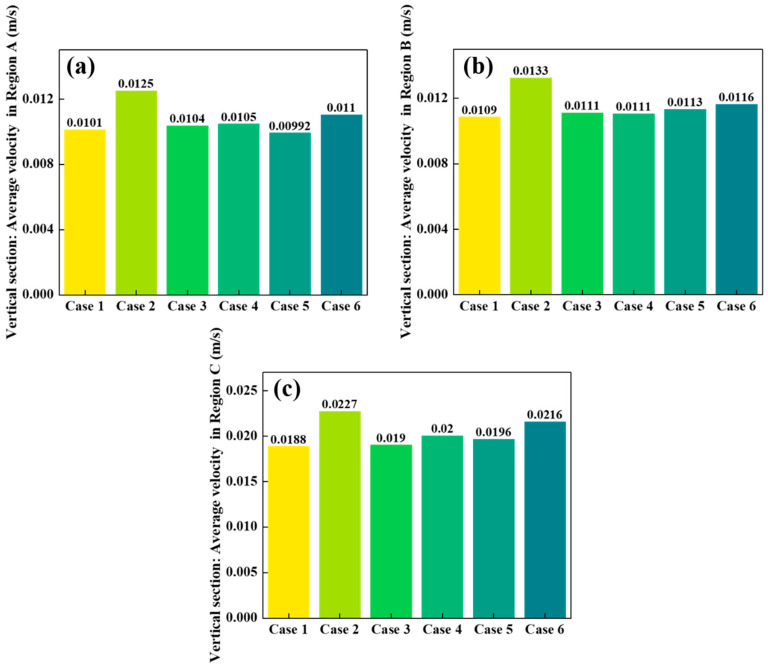
Comparison of particle descent velocities in radially divided regions of the vertical shaft furnace section: (**a**) Region A, (**b**) Region B, (**c**) Region C.

**Figure 7:**
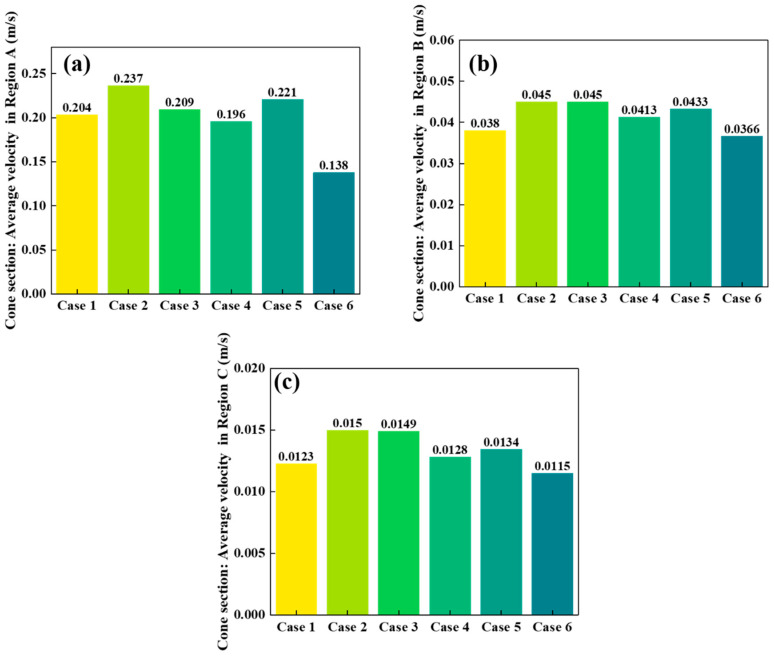
Comparison of particle descent velocities in radially divided regions of the conical section of the shaft furnace: (**a**) Region A, (**b**) Region B, (**c**) Region C.

**Figure 8:**
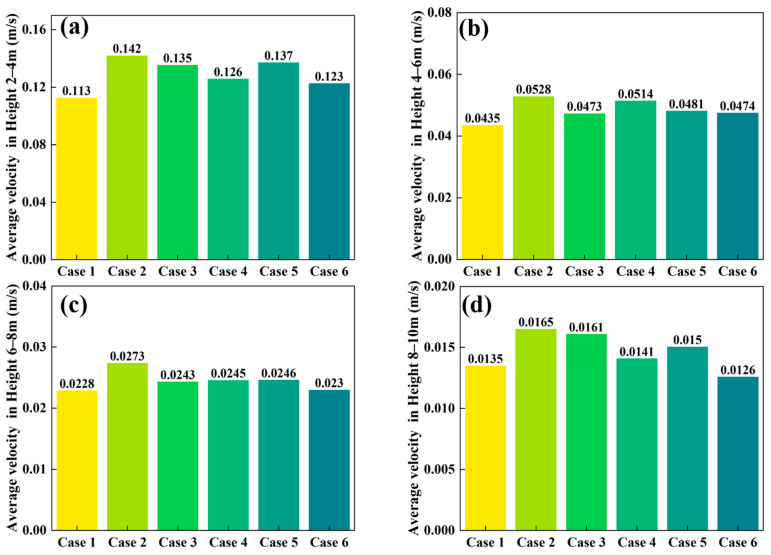
Comparison of particle descent velocities in longitudinally divided regions of the shaft furnace: (**a**) 2–4 m, (**b**) 4–6 m, (**c**) 6–8 m, (**d**) 8–10 m.

Figure 6, Figure 7 and Figure 8 compare the average particle descent velocities in different regions of the shaft furnace under Cases 1–6. Overall, Case 2 exhibits the highest velocities across most regions, which corroborates the discharge trends shown in Figure 5 and confirms its superior flowability.

In the vertical section (Figure 6), the average velocities in Regions A and B are generally low (around 0.010–0.013 m/s), while Region C shows significantly higher values (0.01884–0.02271 m/s). This indicates that particles in the central region descend much faster than those near the walls, consistent with funnel-flow behavior. Among all cases, Case 2 again yields the highest velocities, with Region C reaching 0.02271 m/s, demonstrating the strong effect of distributor geometry on accelerating central flow.

In the conical section (Figure 7), a pronounced velocity gradient is observed among the three regions. Region A exhibits the highest velocities, ranging from 0.13763 to 0.23656 m/s, due to the converging geometry near the outlet. Region B velocities remain moderate (0.03663–0.04501 m/s), while Region C values are the lowest (0.01150–0.01497 m/s). Notably, Case 2 again achieves the largest descent velocity in Region A (0.23656 m/s), indicating enhanced flow through the central discharge pathway.

In the longitudinally divided zones (Figure 8), particle velocity decreases with increasing height. At 2–4 m, average velocities are relatively high (0.11256–0.14192 m/s), but they decrease to 0.04349–0.05281 m/s at 4–6 m, 0.02285–0.02734 m/s at 6–8 m, and 0.01257–0.01651 m/s at 8–10 m. This trend reflects the downward propagation of motion from the outlet upwards. Across all heights, Case 2 consistently maintains the highest velocities, highlighting its favorable impact on the overall flow regime.

Taken together, the results in Figure 6, Figure 7 and Figure 8 demonstrate that Case 2 not only accelerates particle descent in both vertical and conical sections but also promotes faster flow propagation throughout the furnace height. These findings are consistent with the discharge patterns in Figure 5 and the velocity fields in Figure 4, further confirming that optimizing distributor geometry plays a decisive role in enhancing furnace flowability and mitigating flow non-uniformity.

### 3.2. Analysis of the Effects on Particle Flow Trajectories

In order to observe more clearly the impact of the wall angle of the shaft furnace on the flow pattern of pellet particles, two kinds of colors of spherical particles, red and blue, were used to distinguish different layers of pellet. The pellet fell into the shaft furnace under the effect of gravity, and after all particles were free of relative motion, the discharge port was opened.

**Figure 9:**
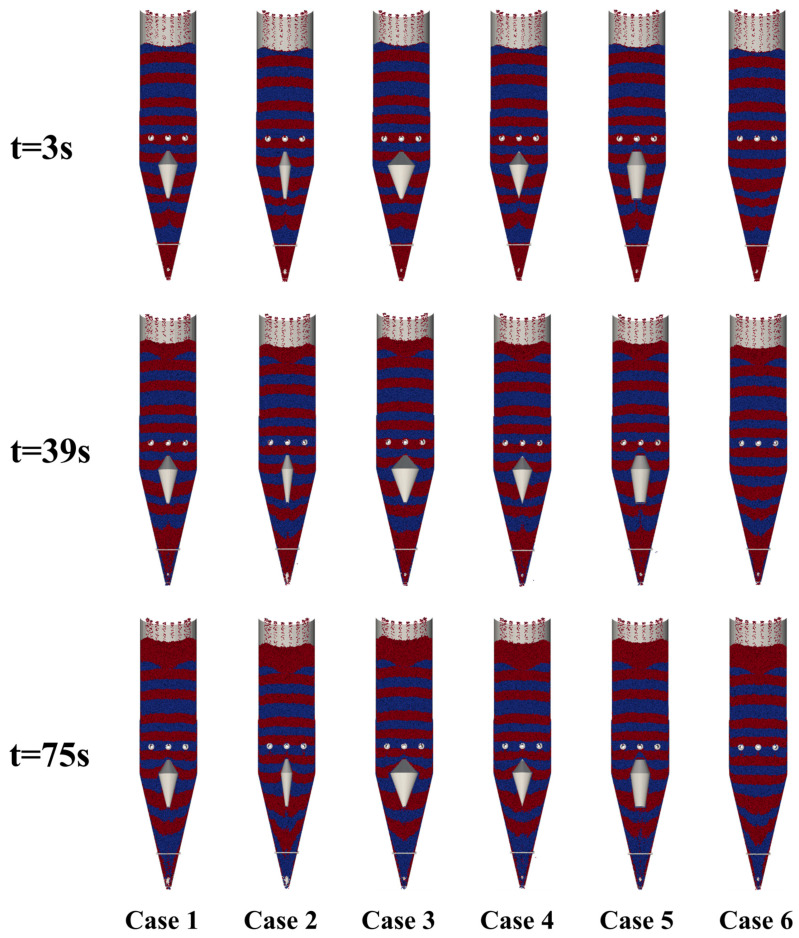
Snapshots of particle flow within the shaft furnace at different times.

Figure 9 presents snapshots of particle flow within the shaft furnace at different times. In the absence of a flow distributor, particles in the central region descend more rapidly, forming a typical V-shaped funnel flow. With the introduction of a flow distributor, the flow pattern transforms into a W-shape or a more uniform distribution. The device slows the descent of central particles, increases the participation of peripheral particles, and enhances the overall uniformity of particle flow.

### 3.3. Analysis of the Effects on Particle Force Conditions

**Figure 10:**
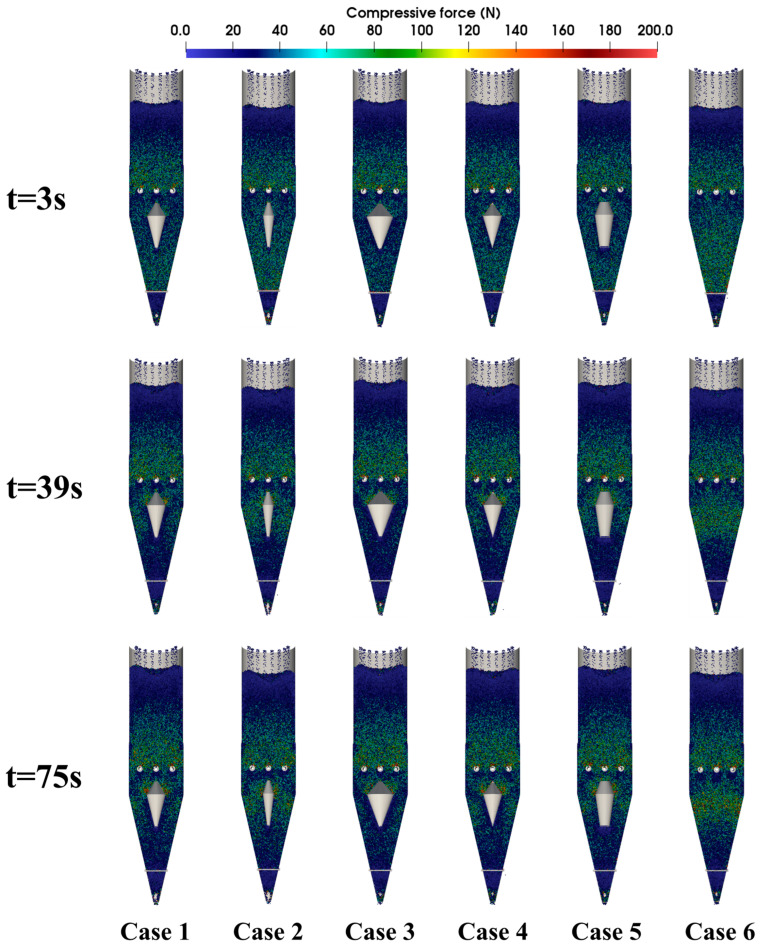
Contour maps of particle compressive force distribution within the shaft furnace at different times.

Figure 10 presents contour maps of particle compressive force distribution within the shaft furnace at different times. At t = 3 s, the discharge has just begun, and pellets experiencing higher compressive forces are concentrated in the lower and middle regions of the furnace. At t = 39 s, the compressive forces are concentrated in the central region, and the high-compression zones in Case 3 are noticeably reduced, indicating that increasing the diameter of the flow distributor helps improve the force conditions of pellets within the furnace.

**Figure 11:**
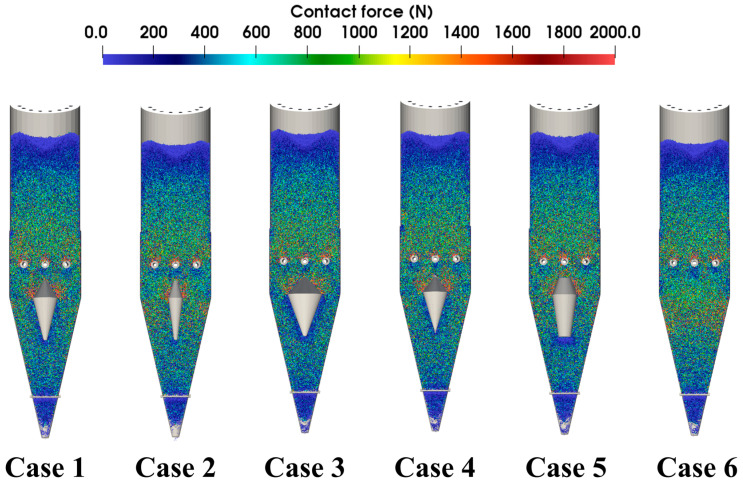
Force chain distribution within the shaft furnace at t = 61.5 s.

Figure 11 shows the force chain distribution within the furnace at t = 61.5 s, which exhibits the same trend as Figure 10, i.e., the interaction forces among particles near the flow distributor in Case 3 are smaller and more evenly distributed. This suggests that an enlarged distributor diameter can alleviate excessive force concentration and lower the risk of local structural instabilities.

**Figure 12:**
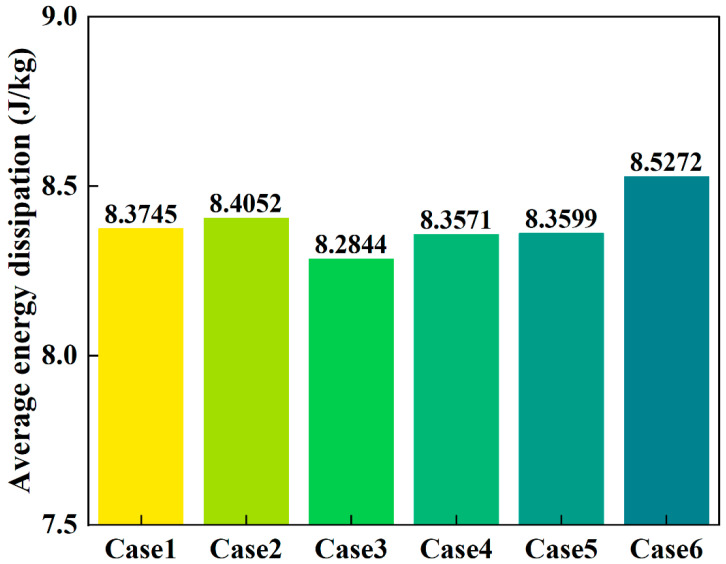
Statistics of average energy dissipation.

Figure 12 presents the averaged energy dissipation caused by particle collisions throughout the entire discharge process within the shaft furnace. It can be observed that the average energy dissipation in Case 3 is the lowest (8.2844 J/kg), whereas Case 6 exhibits the highest value (8.5272 J/kg). This indicates that the use of a flow distributor can reduce the average energy dissipation within the furnace, thereby decreasing the likelihood of pellet breakage. Conversely, the higher average energy dissipation in Case 2 compared to Case 1 suggests that reducing the diameter of the flow distributor increases the probability of pellet fragmentation within the furnace.

Taken together, these results highlight a trade-off between flow uniformity and particle mechanical stability: while smaller-diameter distributors enhance flow efficiency, they can intensify particle collisions and raise the risk of breakage, whereas larger-diameter distributors mitigate compressive force concentration and energy dissipation but may reduce discharge efficiency. In practical shaft furnace operation, such trade-offs must be carefully balanced, since excessive pellet degradation would generate fines, impair bed permeability, and lower hydrogen utilization efficiency.

Moreover, the observed granular-scale mechanisms can be directly linked to furnace-scale performance. Improved flow uniformity contributes to a more stable reduction zone, ensures more homogeneous gas–solid contact, and thereby enhances both metallization efficiency and hydrogen utilization. Conversely, excessive energy dissipation and pellet breakage would lead to fines generation and impaired permeability, which reduce gas flow distribution, lower metallization in the furnace core, and increase overall energy consumption. These interpretations underline that optimizing diverter geometry is not only a matter of mechanical flow regulation but also a key factor for improving the operational efficiency of hydrogen-based shaft furnaces.

## 4. Conclusions

In this study, the impact of different diverter device shapes and sizes on particle flow behavior inside a hydrogen-based direct reduction shaft furnace was investigated using the Discrete Element Method (DEM). This study focused on understanding how varying the design of the diverter device, particularly its size and geometry, influences the uniformity of particle flow, which is crucial for optimizing furnace performance.

(1) The results demonstrate that the introduction of a diverter device significantly improves the flow distribution of particles within the furnace. Specifically, the diverter device helps to mitigate the funnel flow phenomenon observed in the center of the furnace, improving the uniformity of particle descent and enhancing the overall flow behavior. The geometric parameters of the diverter device, such as its radial width and the top/bottom diameter, play a critical role in controlling particle velocity and promoting a more homogeneous flow.
(2) In particular, Case 2, with a smaller central diameter, produced the most favorable dynamics, resulting in faster particle discharge and a more uniform descent compared to other cases. However, this configuration also exhibited relatively higher energy dissipation, implying a potential risk of pellet fragmentation. In contrast, larger distributor diameters (e.g., Case 3) reduced compressive force concentration and lowered average energy dissipation (8.28 J/kg), but with slightly reduced discharge efficiency. These findings highlight the trade-off between flow uniformity and particle integrity.
(3) The results demonstrate that optimizing diverter geometry can provide practical guidance for improving flow behavior in hydrogen-based shaft furnaces, which is essential for stable operation and enhanced process performance.

This work is based on cold-state DEM simulations and does not couple gas flow or reduction reactions. As such, the results reflect only the mechanical aspects of particle motion. Future studies will integrate DEM with CFD and reaction kinetics to establish quantitative links between flow behavior, metallization degree, and hydrogen utilization efficiency.

## Figures and Tables

**Table 1:** Details of shaft furnace region divisions.

	Radial Width, mm	Longitudinal Height, mm
Region A	566	24,000
Region B	1276	24,000
Region C	1276	24,000
Sections 1–12	5670	2000

**Table 2:** Material properties and simulation parameters [13,14,22].

Parameters		Pellet	Wall
Density, ρ (kg/m^3^)		3425	7850
Young’s modulus, E (MPa)		20	30
Poisson’s ratio, υ (-)		0.25	0.3
Restitution coefficient, e (-)	Pellet	0.4	0.35
Static friction, μ_s_ (-)	Pellet	0.5	0.4
Rolling friction, μ_r_ (-)	Pellet	0.2	0.4
Time step, Δt	1 × 10^−5^		

**Table 3:** Variations in the Shape and Dimensions of Internal Flow Distributors in the Shaft Furnace.

	Detailed Description
Case 1	Baseline (central diameter 2000 mm; top/bottom diameter 300 mm)
Case 2	Reduce the central diameter of the flow distributor to 1200 mm
Case 3	Increase the central diameter of the flow distributor to 2800 mm
Case 4	Reduce the top/bottom diameter of the flow distributor to 10 mm
Case 5	Increase the top/bottom diameter of the flow distributor to 1000 mm
Case 6	Remove the flow distributor

## Data Availability

The data presented in this study are available on request from the corresponding authors. The data are not publicly available due to legal and privacy.

## References

[1] Holappa L. (2020). A General Vision for Reduction of Energy Consumption and CO_2_ Emissions from the Steel Industry. Metals.

[2] Ariyama T., Takahashi K., Kawashiri Y., Nouchi T. (2019). Diversification of the Ironmaking Process Toward the Long-Term Global Goal for Carbon Dioxide Mitigation. J. Sustain. Met..

[3] Zhou F., Peng D., Li K., Conejo A.N., Liao H., Xiong Z., Li D., Zhang J. (2024). Coke behavior with H2O in a hydrogen-enriched blast furnace: A review. Int. J. Miner. Metall. Mater..

[4] Wang M., Li Y., Li J., Wang Z. (2021). Green process innovation, green product innovation and its economic performance improvement paths: A survey and structural model. J. Environ. Manag..

[5] Weigel M., Fischedick M., Marzinkowski J., Winzer P. (2016). Multicriteria analysis of primary steelmaking technologies. J. Clean. Prod..

[6] Pei M., Petäjäniemi M., Regnell A., Wijk O. (2020). Toward a fossil free future with HYBRIT: Development of iron and steelmaking technology in Sweden and Finland. Metals.

[7] Sohn H.Y. (2019). Review of fluid-solid reaction analysis—Part 3: Complex fluid-solid reactions. Can. J. Chem. Eng..

[8] Hamadeh H., Mirgaux O., Patisson F. (2018). Detailed modeling of the direct reduction of iron ore in a shaft furnace. Materials.

[9] Yu Y., Saxén H. (2010). Experimental and DEM study of segregation of ternary size particles in a blast furnace top bunker model. Chem. Eng. Sci..

[10] Shi Y., Zhu D., Pan J., Guo Z., Lu S., Xu M. (2022). Improving hydrogen-rich gas-based shaft furnace direct reduction of fired hematite pellets by modifying basicity. Powder Technol..

[11] Shao L., Zhang X., Zhao C., Qu Y., Saxén H., Zou Z. (2021). Computational analysis of hydrogen reduction of iron oxide pellets in a shaft furnace process. Renew. Energy.

[12] Bhattacharya T., McCarthy J. (2014). Chute flow as a means of segregation characterization. Powder Technol..

[13] Liao H., Zong Y., Li K., Bu Y., Bi Z., Jiang C., Liu Z., Zhang J. (2023). Influence of lump ore ratio and shapes on the particle flow behavior inside the direct reduction shaft furnace by discrete element modeling model. Steel Res. Int..

[14] Liao H., Zong Y., Li K., Bi Z., Jiang C., Liu Z., Zhang J., Zhang C. (2023). Influence of Wall Angle of Direct Reduction Shaft Furnace on Particle Flow Pattern by DEM Modeling. Steel Res. Int..

[15] Turkdogan E., Vinters J. (1971). Gaseous reduction of iron oxides: Part I. Reduction of hematite in hydrogen. Met. Trans..

[16] Zieliński J., Zglinicka I., Znak L., Kaszkur Z. (2010). Reduction of Fe_2_O_3_ with hydrogen. Appl. Catal. A Gen..

[17] Pineau A., Kanari N., Gaballah I. (2006). Kinetics of reduction of iron oxides by H_2_: Part I: Low temperature reduction of hematite. Thermochim. Acta.

[18] Pineau A., Kanari N., Gaballah I. (2007). Kinetics of reduction of iron oxides by H_2_: Part II. Low temperature reduction of magnetite. Thermochim. Acta.

[19] Zhao Z., Tang J., Chu M., Wang X., Zheng A., Wang X., Li Y. (2022). Direct reduction swelling behavior of pellets in hydrogen-based shaft furnaces under typical atmospheres. Int. J. Miner. Met. Mater..

[20] Lin H.-Y., Chen Y.-W., Li C. (2003). The mechanism of reduction of iron oxide by hydrogen. Thermochim. Acta.

[21] Cundall P.A., Strack O.D. (1979). A discrete numerical model for granular assemblies. Géotechnique.

[22] Boechat F.O., de Carvalho R.M., Tavares L.M. (2018). Simulation of mechanical degradation of iron ore pellets in a direct reduction furnace. KONA Powder Part. J..

[23] Da Costa A.R., Wagner D., Patisson F. (2013). Modelling a new, low CO_2_ emissions, hydrogen steelmaking process. J. Clean. Prod..

[24] Kuang S., Li Z., Yu A. (2018). Review on modeling and simulation of blast furnace. Steel Res. Int..

[25] Zhou H., Wu S., Kou M., Luo Z., He W., Zou Z., Shen Y. (2018). Discrete Particle Simulation of Solid Flow in a Large-Scale Reduction Shaft Furnace with Center Gas Supply Device. ISIJ Int..

[26] Tian X., Zhou H., Xia T., Qin Z.-T., Guo H.-D., Sun D.-W., Kou M.-Y., Wu S.-L. (2025). Influence of diversion cone structure on inner characteristic in hydrogen-enriched shaft furnace: A DEM study. J. Iron Steel Res. Int..

[27] Zhou Z.Y., Zhu H.P., Yu A.B., Wright B., Zulli P. (2008). Discrete particle simulation of gas–solid flow in a blast furnace. Comput. Chem. Eng..

[28] Yu Y., Saxén H. (2012). Flow of Pellet and Coke Particles in and from a Fixed Chute. Ind. Eng. Chem. Res..

[29] Liao H., Zong Y., Li K., Bi Z., Jiang C., Zhang J., Ren S. (2022). Effect of outlet characteristics and particle properties on the flow characteristics inside conical hoppers. Met. Res. Technol..

